# Isolated myeloid sarcoma as the first manifestation of acute myeloid leukemia: a case study

**DOI:** 10.1002/ccr3.1175

**Published:** 2017-09-22

**Authors:** Dorothy Mitkowski, Lidia Gil

**Affiliations:** ^1^ Students Scientific Society Poznan University of Medical Sciences Poznan Poland; ^2^ Department of Hematology and Bone Marrow Transplantation Poznan University of Medical Sciences Poznan Poland

**Keywords:** Acute myeloid leukemia, extramedullary, hematopoietic stem cell transplantation, myeloid sarcoma

## Abstract

This case report brings awareness to the diverse extramedullary manifestations of isolated myeloid sarcoma, as well as the importance and difficulties that are associated with establishing a rapid diagnosis and initiating treatment.

## Introduction

Myeloid sarcoma (MS) is a rare disease entity that can present as an isolated extramedullary tumor (EM) of immature granulocytic cells. It was first described in 1812 and later named chloroma by King, due to its green color attributed to the presence of myeloperoxidase enzymes 1, 2. MS has been reported in 2.5–8.0% of patients with acute myeloid leukemia (AML) and occurs concurrently with or at relapse of bone marrow leukemia. It can also be associated with myelodysplastic syndrome, chronic myeloid leukemia, or other myeloproliferative neoplasms and very rarely with the absence of bone marrow involvement 3. Given the various sites of occurrence, the clinical manifestations of MS are diverse with the signs and symptoms specific to the location at which it occurs 4.

The most common sites of presentation include the skin, lymph nodes, soft tissue, bone, and periosteum; however, numerous other sites have been observed such as the orbit, ovaries, myocardium, and many more 1, 5. Although the optimal timing and treatment of isolated MS has not been yet established, it has been documented that delayed or inadequately treated isolated MS will almost always progress to AML. The median time for which this occurs is 5–12 months 4. Here, we report an unusual case of isolated MS initially presenting as a tumor on the eyelid. In developing this report, we try to illustrate the difficulties associated with establishing a rapid diagnosis and early initiation of treatment.

## Case Report

**Figure 1:**
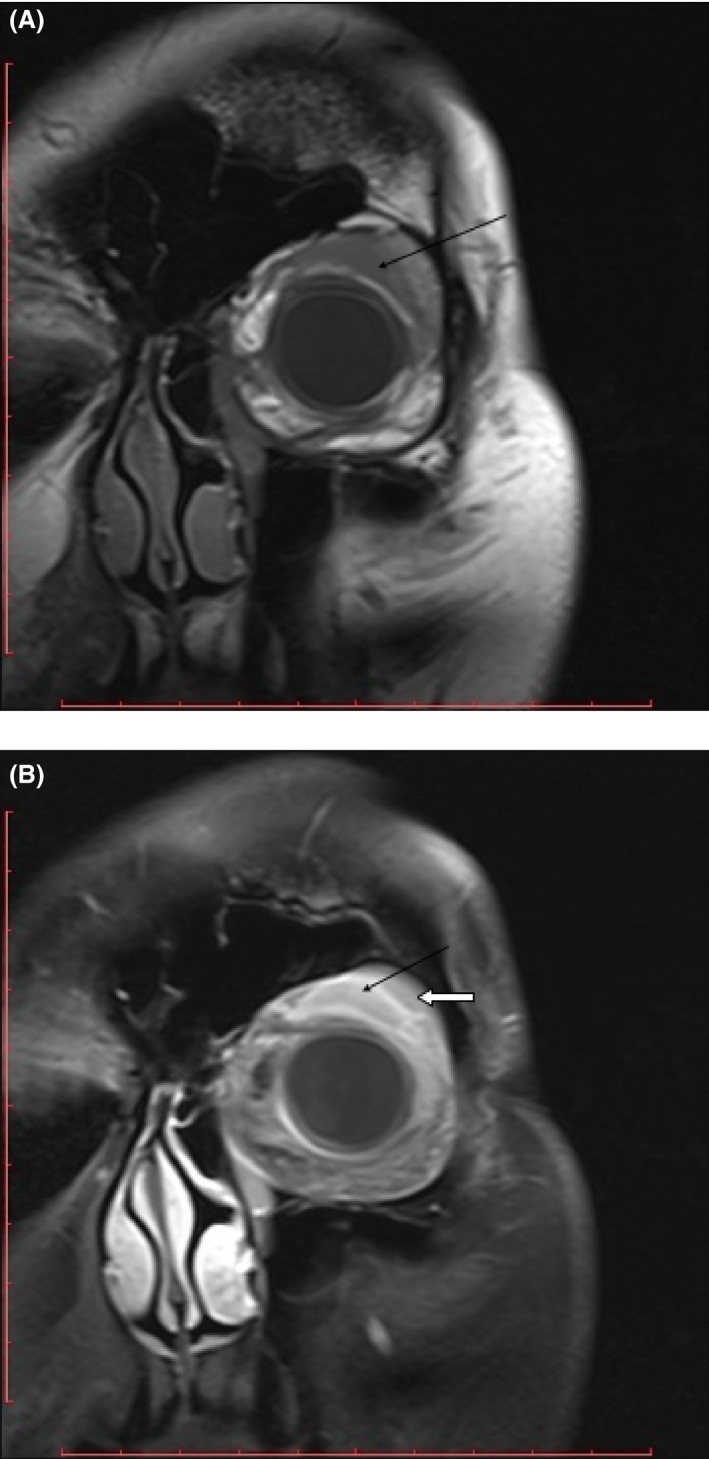
(A) MRI T1 TSE, coronal plane (without contrast). (B) MRI T1 TSE FS, coronal plane (with contrast). Black arrow – pathologic mass; White arrow – lacrimal gland.

**Figure 2:**
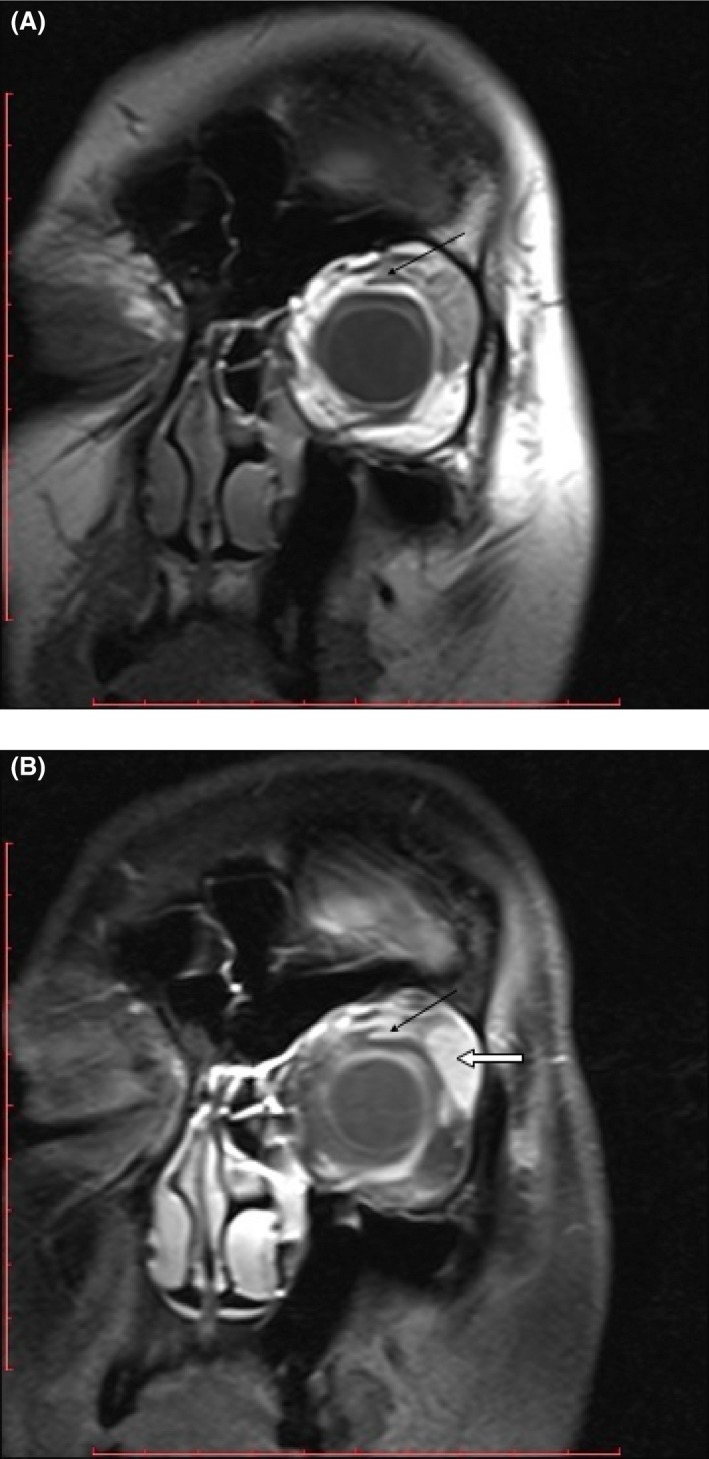
(A) MRI T1 TSE, coronal plane (without contrast). (B) MRI T1 TSE FS, coronal plane (with contrast). Black arrow – residual mass; White arrow – lacrimal gland.

The patient, a 36‐year‐old otherwise healthy, Eastern European, female, presented to her primary care physician, in early February 2015, complaining of nonpainful edema on her left eyelid with a duration of 8 days. She was referred to an ophthalmologist and a laryngologist, who did not discover any abnormalities. A computerized tomography (CT) scan performed at this time yielded negative results. At the end of February 2015, she returned to her ophthalmologist and was diagnosed with having two small, soft tumors, and edema that remained painless on the eyelid. The tumor masses were resected May 25, and histopathological testing revealed MS characterized by infiltration of atypical immature cells with a phenotype of: MPO, CD117+, CD34+, Tdt, LCA positive and CD4, CD15, CD3, S100, HMB45 and CKAE1/3 negative cells, and Ki67 of 50%. Extensive hematological workup, including bone marrow biopsy and immunophenotyping, cytogenetical and molecular study, and positron emission tomography (PET) scan performed in July, all yielded negative results and the patient was discharged from hospital without additional treatment. A month after discharge, the edema reoccured in the same eyelid without any findings in repeated hematological assessment. A magnetic resonance imaging (MRI) scan done at this time revealed a retro‐orbital mass (Fig. 1A and B) and the decision to begin induction chemotherapy with daunorubicin and cytarabine was made on 29 August. A MRI scan conducted after the therapy revealed a remaining retro‐orbital mass, approximately 2 cm in size, and the patient was qualified for re‐induction with Flag‐Ida chemotherapy (fludarabine, cytarabine, idarubicine, granulocyte colony‐stimulating factor) on 29 September in order to treat the refractory disease. A subsequent MRI showed persistence of the mass, which decreased only slightly in size to 16–19 mm; however, a biopsy through the use of neuronavigation systems indicated no neoplastic cells. The patient received consolidation treatment with HDAraC (high‐dose cytarabine) and as a high‐risk patient, she was qualified for allogeneic hematopoietic stem cell transplantation (alloHSCT). No sibling had identical human leukocyte antigens (HLA) to our patient; however, an unrelated 18‐year‐old, male donor, 10:10 match was found on 18 November. Due to infectious complications (bacterial and fungal), the alloHSCT that was originally scheduled for the end of January 2016 was postponed to 9 March. The transplant procedure was performed in complete hematological remission with myeloablative conditioning FluBu4 (fludarabine, busulphan) and prophylaxis of graft versus host and graft rejection with cyclosporine, methotrexate, and ATG. Neutrophil and platelet recovery occurred on day +18. No febrile complications were observed; however, the patient did experience nausea, vomiting, and mucositis III grade according to WHO. She was discharged in very good condition on day +24 after alloHSCT. During post‐transplant period, she developed skin graft‐versus‐host disease grade II on day +90, successfully treated with topical and systemic steroids. A MRI scan done on 29 September 2016, 6 months after alloHSCT (Fig. 2A and B) to assess the persistent mass revealed no changes as compared to the prior MRI scan. Currently, almost 17 months after transplant, the patient remains in complete remission without transplant‐related complications.

## Discussion

The diagnosis of MS often poses a number of challenges, in particular when it develops at an EM site in the absence of bone marrow involvement. The correct diagnosis is made or suspected in only 44% of cases; immunohistochemical stains are usually diagnostic. The most common misdiagnosis is the high‐grade non‐Hodgkin lymphoma (NHL). This occurs due to resulting histopathology that reveals diffusely infiltrating, discohesive cells with numerous lymphocytes characteristic of both MS and NHL. However a differentiating factor is that in MS, the nuclei are slightly smaller with diffuse chromatin 6, 7. Other common errors in diagnosis include thymoma, myeloma, eosinophilic sarcoma, and carcinoma 1. Ophthalmologic manifestation is not an uncommon occurrence among patients with acute leukemia; however, very rarely has it been associated without the involvement of the bone marrow. Retinal lesions are among the most common locations of leukemia, occurring in up to 69% of patients 8, 9. Although MS can involve any ocular tissue, MS involving the eyelid is less common and is usually found secondary to orbital involvement 10, 11. Despite a case reported of MS involving the eyelid and caruncles as the first sign of AML relapsing after bone marrow transplant, there remain limited reports about MS in the eyelid as the initial manifestation of AML, such as this case 8.

As histopathology commonly leads to misdiagnosis, adversely implying incorrect treatment, immunophenotyping, and immunohistochemistry are crucial for making an accurate diagnosis 12, 13. The most commonly expressed marker is CD68/KPI followed by MPO 4. Other common markers include CD4, CD15, CD30, CD34, CD56, CD99, CD117, and Tdt; albeit our patient only expressed CD34, CD117, and Tdt 3. Expression of CD 117, a transmembrane protein receptor encoded by the c‐kit proto‐oncogene, has been associated with poorer outcomes 11. Immunohistochemistry for antimyeloperoxidase (MPO) has been shown to be the most useful and sensitive marker in differentiating between myeloid and nonmyeloid cells. MPO staining is often positive in the malignant cells of EM, allowing for a quick and effective way to rule out other tumors. The Leder stain should be considered to confirm myelocytic differentiation as it has been consecutively helpful in establishing the diagnosis without the involvement of the bone marrow 4. Despite the essential role of immunophenotyping and immunohistochemistry in establishing diagnoses, the absence of certain markers does not exclude MS, as witnessed in our patient.

The most common cytogenic abnormality associated with EM involvement at both initial presentation and at relapse is the t(8;21) translocation 4. Furthermore, this translocation has been found to be more common in childhood or in MS involving the orbit 2. In our case, there were no genetic abnormalities observed.

Once the correct diagnosis of MS has been made, treatment may be initiated. There are currently no guidelines on whether to begin or delay treatment in patients without the involvement of the bone marrow. This decision also brings in the ethical component of autonomy versus beneficence, as the patient generally feels well without bone marrow involvement. Despite this, it has been documented that delayed or inadequately treated isolated MS will almost always progress to AML, with a median time of 5–12 months 4.

There exist a variety of treatment options including chemotherapy, radiotherapy (RT), surgical excision, HSCT, or any combination of these treatments. The standard treatment for isolated MS is similar to that of classic AML 14. In patients with isolated MS, treatment with AML‐based induction regimens had complete remission rates comparable to those with AML without MS and they prolonged disease‐free survival from 3.5 to 16 years 3. In a study done by Lan et al., patients undergoing chemotherapy had significantly longer survival time compared to those who did not (*P* = 0.0009). Furthermore, systemic chemotherapy has been shown to slow the rate of progression in patients with isolated MS to AML (42%) compared to patients that received localized treatment only (88–100%) 14.

In our practice, our treatment approach combined chemotherapy, surgical excision, and alloHSCT. Induction chemotherapy with cytarabine and daunorubicine has been reported to induce complete remission in 65% to 75% of patients 15, 16. There has been some evidence suggesting that cytarabine containing regimens prolonged disease‐free survival compared to patients that were initially misdiagnosed and treated with agents used to treat lymphoma, sarcoma, or multiple myeloma 3. Tsimberidou et al. found that 16 patients with isolated MS that were treated with cytarabine contained regimens had a longer event‐free survival (*P* = 0.08).

As a high‐risk patient, our patient was qualified for alloHSCT, which has been shown to benefit the overall survival and disease‐free survival 3, 17, 18, 19, 20, 21, 22.

The precise role of RT in addition to systemic chemotherapy has not been yet established 3. In our patient, RT was not used during treatment. This was because the disease was resistant to first‐line chemotherapy with daunorubicin and cytarabine. In such cases, it is our centre's policy to offer alloHSCT. Bakst et al. considers the use of RT in patients with isolated MS with inadequate response to chemotherapy and recurrence after alloHSCT. A recent study suggested that RT may prolong failure‐free survival but not overall survival in patients presenting with isolated MS. When RT was used in combination with chemotherapy in three patients, none of these patients progressed to AML 23. Despite this, there remain insufficient studies addressing the role of RT and whether this protocol results in a superior overall outcome compared with chemotherapy alone 4.

## Conclusion

This case illustrates the challenges associated with developing a rapid diagnosis with early initiation of treatment. Accurate diagnosis of isolated MS requires a multifactorial approach including histopathology, immunophenotyping, immunohistochemistry, cytogenic abnormalities, and also clinical suspicion. Although the ophthalmologist does not play a direct role in the treatment of isolated MS, prompt recognition of ocular manifestation as a sign of EM is imperative in the rapid initiation of treatment. Systemic chemotherapy similar to that of leukemic AML is currently the standard treatment. RT and alloHSCT have been found to increase the survival rates in patients with isolated MS; however, newer and larger prospective studies are required in order to obtain a better understanding of the optimal timing and treatment of isolated MS.

## Authorship

DM: involved in writing the manuscript. LG: had the whole supervision of the case. Both authors approved the final version of the case report for submission to the Clinical Case Reports.

## Conflict of Interest

None declared.
